# Impaired Skeletal Muscle Regeneration in the Absence of Fibrosis during Hibernation in 13-Lined Ground Squirrels

**DOI:** 10.1371/journal.pone.0048884

**Published:** 2012-11-14

**Authors:** Eva Andres-Mateos, Rebeca Mejias, Arshia Soleimani, Brian M. Lin, Tyesha N. Burks, Ruth Marx, Benjamin Lin, Richard C. Zellars, Yonggang Zhang, David L. Huso, Tom G. Marr, Leslie A. Leinwand, Dana K. Merriman, Ronald D. Cohn

**Affiliations:** 1 McKusick-Nathans Institute of Genetic Medicine, Johns Hopkins University School of Medicine, Baltimore, Maryland, United States of America; 2 Department of Radiation Oncology and Molecular Radiation Sciences, Johns Hopkins School of Medicine, Baltimore, Maryland, United States of America; 3 Department of Molecular and Comparative Pathobiology, Johns Hopkins University School of Medicine, Baltimore, Maryland, United States of America; 4 Hiberna Corporation, Boulder, Colorado, United States of America; 5 Department of Molecular, Cellular, and Developmental Biology, University of Colorado, Boulder, Colorado, United States of America; 6 Department of Biology and Microbiology, University of Wisconsin, Oshkosh, Wisconsin, United States of America; 7 Department of Neurology, Johns Hopkins University School of Medicine, Baltimore, Maryland, United States of America; 8 Department of Pediatrics, Johns Hopkins University School of Medicine, Baltimore, Maryland, United States of America; UNC Eshelman School of Pharmacy, United States of America

## Abstract

Skeletal muscle atrophy can occur as a consequence of immobilization and/or starvation in the majority of vertebrates studied. In contrast, hibernating mammals are protected against the loss of muscle mass despite long periods of inactivity and lack of food intake. Resident muscle-specific stem cells (satellite cells) are known to be activated by muscle injury and their activation contributes to the regeneration of muscle, but whether satellite cells play a role in hibernation is unknown. In the hibernating 13-lined ground squirrel we show that muscles ablated of satellite cells were still protected against atrophy, demonstrating that satellite cells are not involved in the maintenance of skeletal muscle during hibernation. Additionally, hibernating skeletal muscle showed extremely slow regeneration in response to injury, due to repression of satellite cell activation and myoblast differentiation caused by a fine-tuned interplay of p21, myostatin, MAPK, and Wnt signaling pathways. Interestingly, despite long periods of inflammation and lack of efficient regeneration, injured skeletal muscle from hibernating animals did not develop fibrosis and was capable of complete recovery when animals emerged naturally from hibernation. We propose that hibernating squirrels represent a new model system that permits evaluation of impaired skeletal muscle remodeling in the absence of formation of tissue fibrosis.

## Introduction

Skeletal muscle atrophy can occur as a consequence of immobilization and/or starvation in the majority of vertebrates studied. The resulting loss of muscle mass in these conditions involves a general acceleration of proteolysis and a decrease in protein synthesis [1]. Loss of skeletal muscle mass increases morbidity, mortality, and the incidence of pathologic fractures and hospitalization [2], [3], [4]. Interestingly, hibernating mammals are protected against the loss of muscle mass despite long-term inactivity and anorexia during their winter dormant period (torpor and interbout arousals; see materials and methods section for specific terminology of hibernation) [5], [6], [7], [8], [9]. Therefore, hibernating animals are a natural model system to study protective mechanisms against skeletal muscle atrophy after extended periods of inactivity and starvation.

**Figure 1:**
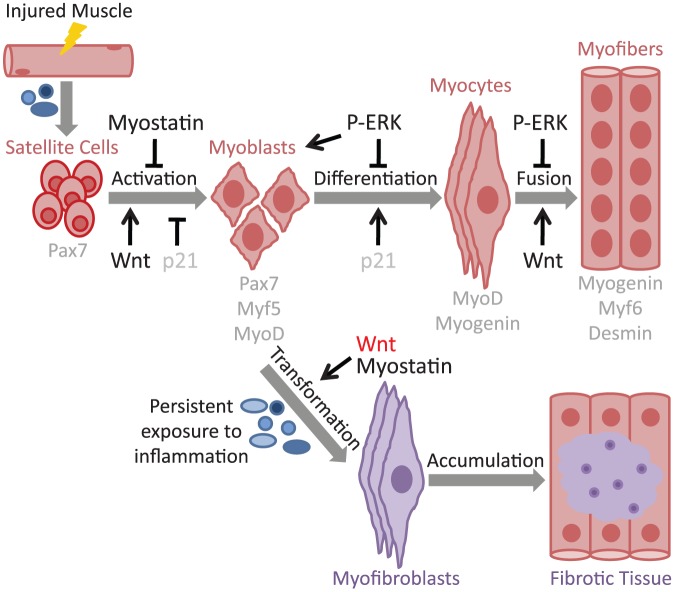
Schematic diagram of myogenic and fibrosis regulatory pathways in mammals. After skeletal muscle damage occurs, cytokines and growth factors are released from the injured blood vessels and from infiltrating inflammatory cells. As a result of these coordinated events, satellite cells undergo extensive proliferation upon activation. Activated satellite cells (also called MCPs or myoblasts) will differentiate into myotubes and fuse together with either damaged myofibers or form new myofibers, while some will undergo self-renewal to restore the satellite cell pool. If inflammatory cell infiltration and fibroblast activation persist, the aberrant tissue repair response will produce a non-functional mass of fibrotic tissue. Myogenic regulatory pathways and factors are indicated. In hibernating squirrels, activation of Wnt signaling pathway (Wnt) does not favor fibrosis formation as has been shown in other mammals (in red).

Skeletal muscle is a highly plastic tissue with remarkable regenerative capacity after injury. Muscle regeneration depends on resident skeletal muscle stem cells called satellite cells, located between the sarcolemma and basement membrane of muscle fibers [10]. After skeletal muscle damage occurs, cytokines and growth factors are released from the injured blood vessels and from infiltrating inflammatory cells [11]. The cytokines promote not only the migration of the inflammatory cells to the site of injury, but also mediate proliferation and cell survival of several cell types. The inflammatory cells are responsible for the phagocytosis of cell debris [12]. As a result of these coordinated events, satellite cells are activated and undergo extensive proliferation upon activation. Activated satellite cells will differentiate into myotubes and fuse together with either damaged myofibers or form new myofibers, while some will undergo self-renewal to restore the satellite cell pool (Fig. 1) [13]. Efficient muscle repair also requires the migration and proliferation of fibroblasts in order to produce additional extracellular matrix (ECM) components, which will act as a scaffold for the new myofibers. Finally, a new vascular network is established and maturation of the newly formed muscle fibers occurs. However, if inflammatory cell infiltration and fibroblast activation persist, an aberrant tissue repair response will produce a non-functional mass of fibrotic tissue. Understanding the mechanisms involved in abnormal muscle repair is necessary to develop strategies to combat the decline evident in a variety of neuromuscular conditions such as aging muscle (sarcopenia) and progression of numerous muscular dystrophies. Therefore, multiple studies have focused on identifying ECM proteins, cytokines, growth factors and the downstream signaling pathways involved in aberrant muscle regeneration and fibrotic tissue formation [14], [15], [16].

The role of satellite cells in the protection against atrophy, or the regeneration process in hibernating animals has not been studied to date. Here we show that satellite cells are not a major contributing factor in the maintenance of skeletal muscle mass after the extended periods of inactivity that occur during hibernation. However, we demonstrate that satellite cells are, in fact, necessary for skeletal muscle regeneration after cardiotoxin injection in hibernating 13-lined ground squirrels. Despite slow skeletal muscle regeneration during hibernation, the damaged tissue does not degenerate irreversibly to fibrotic tissue and is capable of complete recovery when animals arouse from torpor in spring. Our data provide evidence that hibernating squirrels represent an animal model to study the preservation of skeletal muscle in the context of impaired muscle remodeling without the formation of fibrosis.

## Results

### Satellite cells are not involved in protection against skeletal muscle atrophy during hibernation

**Figure 2:**
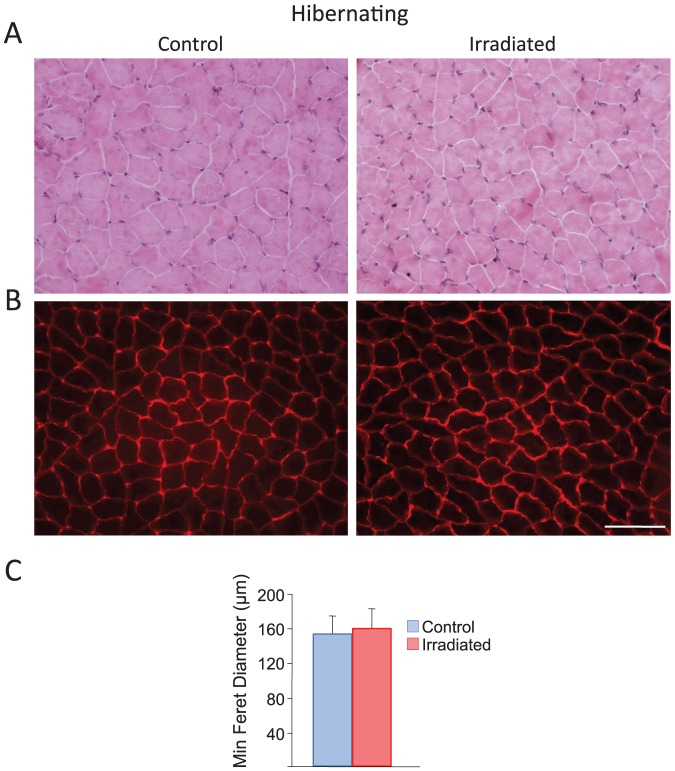
Satellite cells are not involved in maintenance of skeletal muscle during hibernation. Irradiation was used to ablate satellite cells from one quadriceps muscle of hibernating squirrels. **A:** Hematoxylin-eosin (H&E) staining revealed no morphological differences between untreated control (left) and irradiated (right) quadriceps. **B:** Dystrophin immunolabeling outlined myofiber sarcolemmas enabling determination of percentage distribution of minimal Feret's diameter and revealed no effect of satellite cell ablation (scale bar 100 µm). **C:** Mean minimal Feret's diameter (µm) was not significantly different between control and irradiated quadriceps during hibernation (n = 3).

Our laboratory and others have shown that hibernating mammals are protected from muscle atrophy despite prolonged periods of immobilization and starvation [5], [7], [17], [18]. To study the role of satellite cells (SC) in skeletal muscle homeostasis during hibernation, we exposed the right hind-limb from hibernating torpid 13-lined ground squirrels to 25 Gy of radiation to inactivate the SC population of quadriceps muscle [19]. Animals continued in torpor after the irradiation, and were sacrificed 5 weeks after the procedure. Morphological and morphometric analyses showed no differences between irradiated and non-irradiated quadriceps (Fig. 2). These data demonstrate that SC are not actively involved in maintenance of skeletal muscle mass during hibernation.

### Lack of fibrosis development despite impaired active muscle regeneration during hibernation

**Figure 3:**
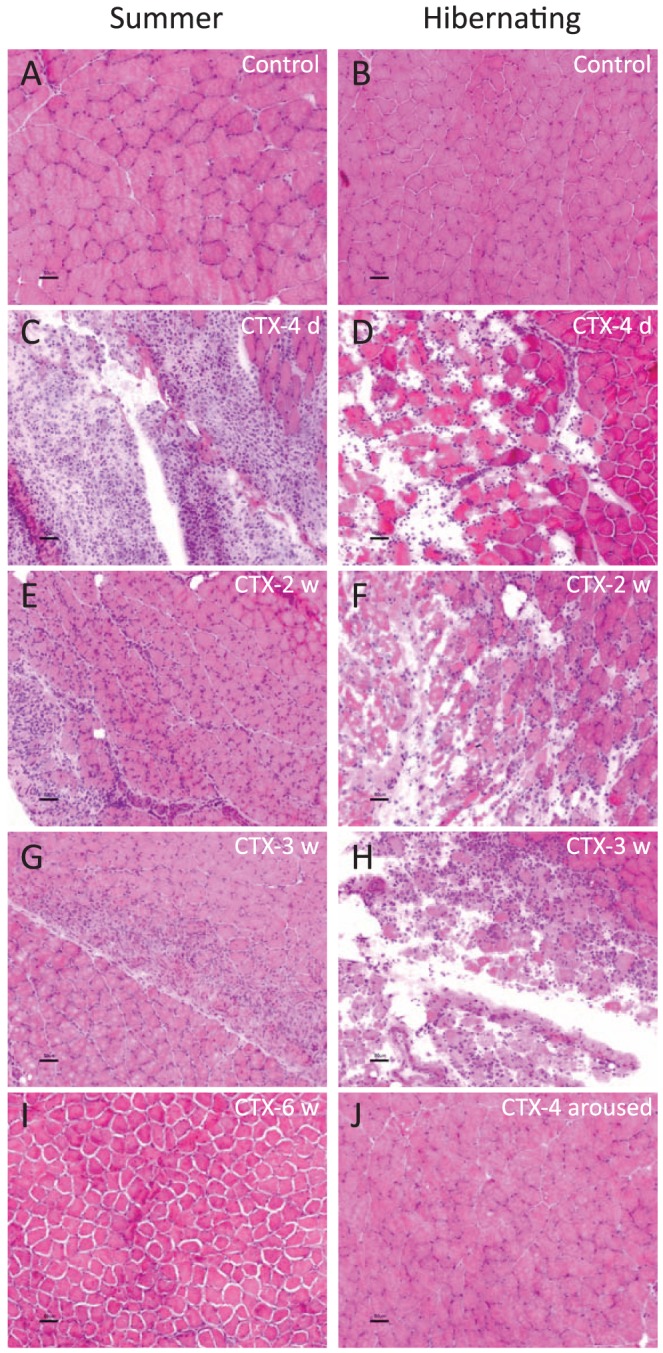
Skeletal muscle regeneration is impaired during hibernation. H&E staining revealed the time course of degeneration, regeneration, and recovery in cardiotoxin-injected gastrocnemius muscles of summer (left) and hibernating (right) squirrels (n = 22; scale bars 50 µm). **A–B:** Uninjured control gastrocnemius from summer (left) and hibernating (right) squirrel illustrating normal myofiber morphology. **C–D**: 4 days after CTX injury, damage and inflammation was evident in both groups. **E–F**: 2 weeks after CTX injury, remodeling was underway in summer, but not hibernating, muscle. **G–H**: 3 weeks after CTX injury, recovery in hibernating muscle still lagged well behind that of summer muscle but displayed no fibrosis. **I**: 6 weeks after CTX injury, summer muscle was fully recovered. **J**: 4 weeks after arousal (following 6–10 weeks of hibernation after CTX injury), muscle achieved full recovery without fibrosis.

**Figure 4:**
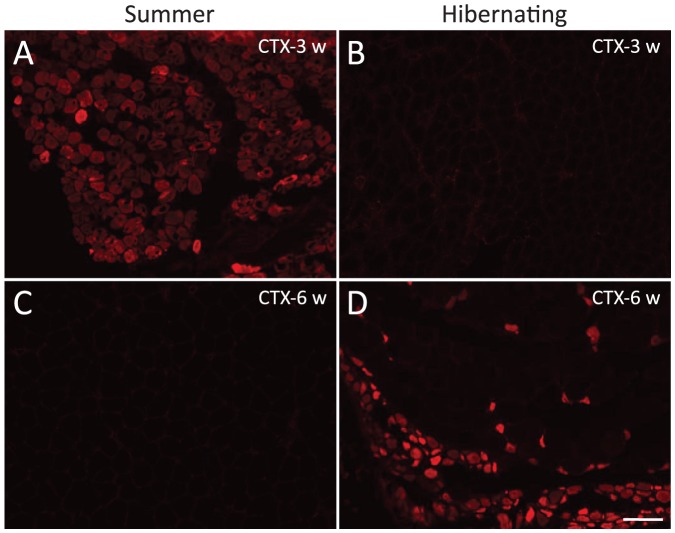
Regenerating fibers do not appear until 6 weeks after cardiotoxin injection in hibernating squirrels. Developmental myosin immunofluorescence in summer (left) and torpid squirrels (right) sacrificed 3 and 6 weeks (w) after the injury. Summer (**A**) but not hibernating squirrels (**B**) have regenerating skeletal muscle fibers 3 weeks after cardiotoxin (CTX) injection. **C:** Muscle of summer squirrels was completely repaired by 6 w after CTX injection. **D:** First regenerating fibers in torpid animals appeared at 6 weeks after the injury (scale bar 100 µm).

**Figure 5:**
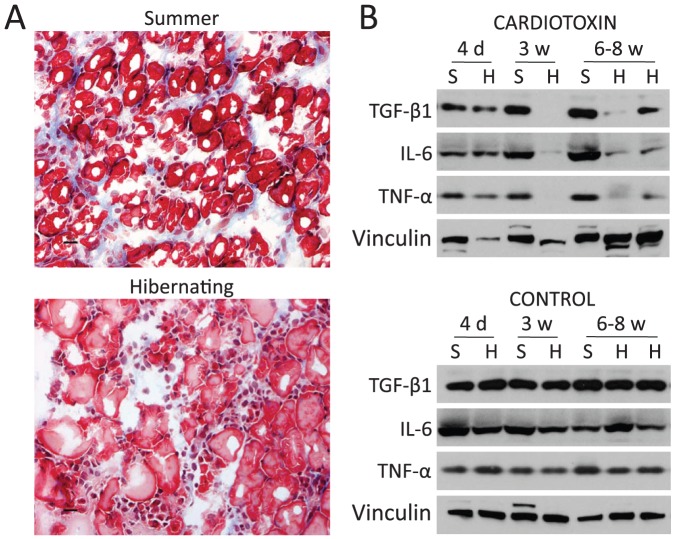
Lack of fibrosis and suppression of inflammatory markers during hibernation. **A:** Masson's trichrome staining showing collagen in blue, from summer and hibernating squirrels 6 weeks after cardiotoxin injury. B: Western blots of gastrocnemius muscle injected with cardiotoxin from summer active (S) and hibernating (H) squirrels using antibodies against fibrosis and inflammatory factors. d = days; w = weeks. Bars represent 20 µm.

To determine whether squirrels maintain the ability to actively regenerate muscle during hibernation, we injured the gastrocnemius muscle by intramuscular administration of the snake venom cardiotoxin (CTX) [20]. Injury during torpor did not interrupt the normal hibernation pattern nor trigger arousal in any of the torpid squirrels. Both seasonally aroused (summer) and hibernating squirrels were sacrificed at 4 days (d), and 1, 2, 3, 6, 8 and 10 weeks (w) after CTX injection. Histological analyses of the gastrocnemius muscle 4 d after injury showed clear signs of muscle injury including macrophage infiltration and degenerating fibers in both summer and hibernating animals (Fig. 3C and Fig. 3D). Regenerating fibers (stained for developmental myosin a marker for regenerating muscle cells) were not present 4 d after injury either in summer (Fig. S1B) or in hibernating animals (data not shown), indicating that regeneration in 13-lined ground squirrels is slower than in other rodents studied [21]. Two to three weeks post-injection, only non-hibernating summer animals showed remodeling, as determined by morphology (Fig. 3E *vs*. 3F) and positive staining of fibers for developmental myosin (Fig. 4 and Fig. S1D). Muscle of summer squirrels was completely repaired and inflammation and macrophage infiltration was completely resolved by 6 w after CTX injection (Fig. 3I and Fig. 4C). In contrast, the first regenerating fibers appeared 6 w after CTX in the muscle of injured hibernating squirrels (Fig. S1A, and Fig. 4D); and muscle remodeling did not occur until 8 w (Fig. S1A and Fig. S1C). In addition to delayed muscle remodeling, hibernating animals also exhibited large areas of inflammation, edema, macrophage infiltration and fatty degeneration (infiltration of adipocytes) that continued 6–10 w after injury (Fig. S1A). In order to visualize the extent of fibrosis, we performed Masson's trichrome staining, which showed absence of connective tissue in hibernating animals 6 w after injury (Fig. 5A). In addition, we found decreased levels of TGF-β1 (transforming growth factor beta 1), a major growth factor related to the formation of fibrosis in skeletal muscle [22] [23] [24], and proinflammatory cytokines TNF-α (tumor necrosis factor-alpha) and IL-6 (interleukin-6), in torpor animals 3–6 w after treatment with CTX. Remarkably, our results showed that unlike any other mammals studied to date, injured skeletal muscle did not form fibrotic scar tissue during hibernation, despite long periods of inflammation and lack of remodeling and decreased TGF-β1 levels may contribute to the protection against formation of fibrotic tissue (Fig. 5, Fig. 3H, and Fig. S1A).

Given the observed delay in muscle remodelling during hibernation, we next examined muscle from squirrels that remained in hibernation for 6–10 weeks after CTX injury and then emerged naturally from hibernation. These squirrels were sacrificed 4 weeks after emergence of torpor and showed complete recovery from the injury without fibrosis (Fig. 3J and Fig. S1E).

Taken together, these data suggest that while active muscle regeneration does not occur during hibernation, injured skeletal muscle is maintained in a non-fibrotic state permissive for muscle regeneration and recovery upon arousal from hibernation, even after longstanding muscle damage.

### Impaired muscle regeneration in hibernation is caused by inhibition of satellite cell activation and myoblast differentiation

In order to elucidate the possible molecular mechanisms underlying the atypical progression of squirrel muscle injury response, we next examined signaling pathways that have previously been shown to play a critical role in muscle regeneration and remodeling in other mammals (Fig. 1).

Muscle regeneration in non-hibernating mammals is characterized by the activation of SC, which leads to proliferation of myogenic precursor cells (MCPs, also called myoblasts) that express Myf5 and MyoD, both of which are required for myogenic determination [25], [26]. Cyclin-dependent kinase inhibitor 1 (p21) and myogenin are then essential for coordination of cell cycle exit and differentiation into myocytes [27], [28]. Lastly, the terminal differentiation process involving fusion of the myocytes to generate myofibers requires Desmin and Myf6 (or MRF4) [29], [30], [31], [32]. Myostatin is a negative regulator of SC activation, regeneration, and muscle growth [33], [34]. In addition, it has been reported that absence of myostatin decreases fibrosis during regeneration [35], [36]. MAPK signaling plays an important role in the regulation of various steps of muscle regeneration. Specifically, ERK signaling has been shown to repress myoblast differentiation [37]. Another critical signaling pathway important for regulating muscle regeneration and fibrotic tissue formation is Wnt signaling [38], [39], [40] (Fig. 1).

**Figure 6:**
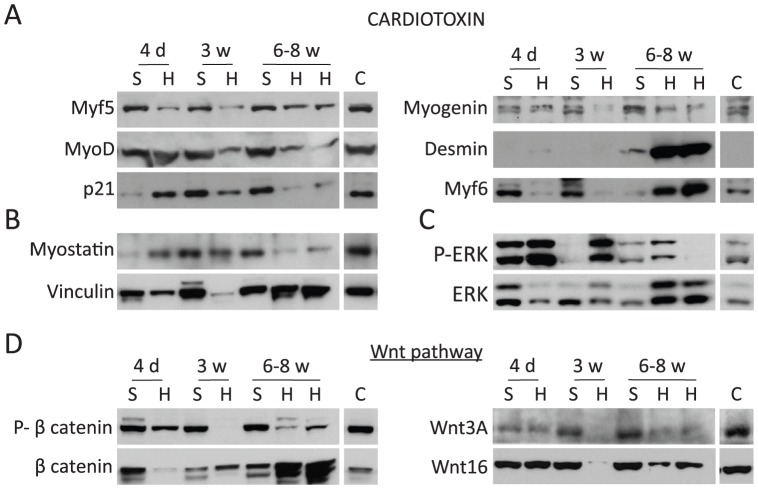
Delayed differentiation of myocytes during regeneration in hibernating squirrels. **A–D:** Western blots of gastrocnemius muscle injected with cardiotoxin from summer active (S) and hibernating (H) squirrels using antibodies against the proteins indicated. For comparison, a representative sample from the contralateral non-injected gastrocnemius is shown (C = Control). P = Phospho; d = days; w = weeks.

We compared protein levels of some of these key mediators from the CTX-injured gastrocnemius from both summer and hibernating squirrels (Fig. 6). The contralateral gastrocnemius was not injected with CTX and so served as an undamaged negative control. These control samples exhibited no significant differences in protein levels between summer and hibernating muscle (Fig. S2A–S2D). Representative samples of the control Westerns blots from non-injected gastrocnemius are included in Fig. 6 for comparison (labeled as C = Control).

Our histological data showed that summer squirrels exhibited a normal regeneration process nearly completed by 3 w. As expected by previous analyses of these pathway components in other rodents (Fig. 1), we saw a decrease of p21 at 4 d, which is important for regulation of the proliferative capacity of MCPs [27] (Fig. 6A). Then, at 3 w, p21 is expressed at higher levels, which is necessary for initiating differentiation of myoblasts to myocytes. Markers of differentiation, Myogenin, MyoD, and Myf6, are expressed at 4 d and 3 w (Fig. 6A), suggesting that the cell machinery is ready to complete the regeneration process at very early time points in summer squirrels. Interestingly, we did not see an increase of desmin levels at 4 d and 3 w after injury in summer squirrels. Because the regeneration process is almost complete at 3 w, we speculate that an increase of desmin may have occurred at an intermediate time-point between 4 d and 3 w. Conversely, in our histological data torpor squirrels showed the first evidence of muscle regeneration at 6 w and muscle remodeling did not occur until 8 weeks after CTX injury. In hibernating gastrocnemius injected with CTX, p21 levels started to decrease at 3 w after injury with a more pronounced decrease at 6–8 w after injury (Fig. 6A). In addition, we detected low levels of Myf5 at 4 d and 3 w followed by an increase at 6–8 w after injury. Taken together, these data suggest that activation of SC in hibernating animals is inhibited at 4 d and at 3 w, and proliferation of MCPs is finally possible at 6–8 w after CTX treatment. In addition, we detected low levels of the differentiation markers, Myogenin, MyoD, and Myf6, 3 w after CTX injection in hibernating animals (Fig. 6A). Late markers of regeneration, Desmin and Myf6, were notably induced at 6–8 w. These results suggest that the muscle differentiation process is also inhibited in hibernating squirrels until 6–8 weeks after injury.

In summer animals, there is a decrease of myostatin levels at 4 d, coinciding with proliferation of MCPs, and then expression recovers when regeneration is nearly completed at 3 w. Interestingly, hibernating squirrels maintained low levels of myostatin for prolonged periods of time (Fig. 6B), suggesting that decreased myostatin signaling may contribute to the protection against formation of fibrotic tissue. Similar to summer squirrels, the lowest levels of myostatin in hibernating animals (6–8 w) overlapped with the proliferation of myoblasts.

In order to determine whether MAPK signaling plays a role in the regulation of muscle regeneration in squirrels, we analyzed ERK signaling pathway, which has been shown to repress myoblast differentiation. Regenerating summer squirrels exhibited evidence for inhibition of ERK signaling, which is necessary for muscle differentiation at 3 w, while no inhibition of ERK signaling was detected until 6–8 w in hibernating squirrels (Fig. 6C). Our results suggest that regulation of ERK signaling activation is one of the molecular mechanisms involved in the delay of muscle regeneration in hibernating animals.

When Wnt ligands are absent, GSK-3β phosphorylates β-catenin, which allow rapid degradation of β-catenin by the proteasome. Upon Wnt activation, GSK-3β is inhibited, allowing non-phosphorylated β-catenin to accumulate and activate target genes involved in the regulation of proliferation and differentiation [41], [42], [43]. Summer squirrels did not reveal evidence for increased canonical Wnt signaling (an increase in the ratio of non-phosphorylated β-catenin to total β-catenin) at any of the observed time points (Fig. 6D). Interestingly, in hibernating squirrels, we detected a clear activation of Wnt signaling at 3 w and 6–8 w after CTX injection that was not mediated by Wnt3A or Wnt16, ligands which have been previously been shown to be involved in muscle proliferation, differentiation and regeneration (Fig. 6D) [38] [39] [44] [45]. These data suggest, that in hibernating animals Wnt signaling activation may regulate the activation of satellite cells and the late differentiation process, as has been shown before in other rodents studied. In summer animals we do not see that regulation, but we cannot rule out that such activation occurs at time points not analyzed.

In summary, our results demonstrate that myogenic regulatory pathways and factors in non-hibernating squirrels behave similarly as described before in other non-hibernating mammals. In contrast, hibernating animals displayed a significant delay in the regeneration process when compared to summer squirrels. This delay can be explained by the inhibition of the satellite cell activation and muscle differentiation, which is regulated by the interplay of p21, myostatin, MAPK, and Wnt signaling pathways.

## Discussion

Prolonged periods of immobilization in humans result in skeletal muscle atrophy, which has a deleterious impact on muscle function and promotes fractures and institutionalization [2], [3], [4]. In contrast, hibernating mammals are naturally protected against skeletal muscle atrophy caused by inactivity and starvation [5], [6], [7], [8], [9]. Our laboratory and others have studied the protective mechanisms against disuse and starvation atrophy in skeletal muscle of hibernating animals [5], [9], [46], [47], [48], [49], [50], [51]. However, whether satellite cells play a role in the maintenance of muscle homeostasis during hibernation is unknown. Here we inactivated the satellite cell population by irradiation [19] and found no associated muscle atrophy, suggesting that preservation of skeletal muscle mass during hibernation is not primarily due to active muscle cell turnover regulated by satellite cells.

Adult skeletal muscle has the remarkable ability to regenerate and remodel in response to various physiologic and non-physiologic stimuli, a process largely attributed to satellite cells [10]. In adult muscle, satellite cells are quiescent under normal physiologic conditions. However, in response to activation signals resulting from various challenges including exercise, injury, and myopathic disorders, satellite cells are activated to repair muscle injury. Efficient muscle regeneration is also thought to play a role in preventing the formation of fibrotic tissue. A subset of these activated cells differentiate into mature muscle cells, while other cells return to the quiescent state during the process of self-renewal [10]. Here we demonstrate that hibernating 13-lined ground squirrels do not actively repair CTX-induced muscle damage, and muscle remodeling is delayed in hibernation compared to remodeling in muscles of euthermic summer animals. In contrast to other mammals and humans where lack of muscle repair is accompanied by fibrosis just after only 2 weeks without proper regeneration [52], [53], hibernating squirrels maintain an afibrotic state that is permissive for muscle regeneration and recovery when the animals arouse from hibernation. These findings are of particular significance as they demonstrate for the first time that delay or absent muscle remodeling does not necessarily proceed toward the fibrotic tissue formation. Understanding the mechanisms by which injured muscle of hibernating squirrels is protected against fibrosis and ultimately achieves full recovery may lead to novel strategies to combat fibrosis associated with impaired muscle regeneration in sarcopenia, muscle injuries and progression of various forms of muscular dystrophy.

In order to gain insights into the underlying molecular mechanisms responsible for these observations, we analyzed markers of inflammation, myogenesis, proliferation and differentiation, and pathways involved in the formation of fibrosis in skeletal muscle. A balanced inflammatory response plays an important role during the process of muscle healing and involves TGF-β1, and cytokines IL-6 and TNF-α particularly during the early inflammatory response [11] [54]. IL-6 and TNF-α have been shown to increase the proliferation of myoblasts but not muscle differentiation [55] [56] [57]. However, persistent presence of pro-inflammatory factors and pro-fibrotic growth factors such as TGF-β1 can lead to aberrant tissue repair resulting in fibrosis [16] [22] [23] [24]. We found low levels of TGF-β1, TNF-α, and IL-6 in torpor animals 3–6 w after treatment with CTX but high presence of macrophages at the same time points. The expression levels of these proteins starts decreasing at 4 d and recovering at 8 w after injury in hibernating animals. Thus, we hypothesize that maintenance of low levels of TGF-β1, TNF-α, and IL-6 contributes to the delayed regeneration response but at the same time protects hibernating skeletal muscle from the formation of tissue fibrosis.

We showed that delayed regeneration in hibernating muscle is caused by inhibition of satellite cells activation and myoblast differentiation, which is regulated by a fine-tuned interplay of p21, myostatin, MAPK, and Wnt signaling pathways. In more detail, we observed delayed p21 decrease and ERK signaling inhibition, activation of Wnt signaling pathway and maintained low levels of myostatin in hibernating animals treated with CTX compared to summer squirrels. p21 is known to be critical for regulation of the proliferative capacity of satellite cells and differentiation of myoblasts into myocytes [27]. ERK signaling has previously been shown to play a role in regulating cell growth and repressing myoblast differentiation [37], [58], [59]. In addition, activation of ERK signaling may also have some beneficial effects on early stages of muscle regeneration [60] and in wound repair [61], [62]. Myostatin is a negative regulator of satellite cell activation, regeneration, and muscle growth [33], [34], [63], [64]. In addition, it has been reported that myostatin inhibition decreases fibrosis after muscle injury, and in genetic models of muscular dystrophy [35], [36], [65], [66], [67], [68]. The effect of myostatin may be due to stimulation of proliferation of myofibroblasts [36]. Based on our results, we propose that down-regulation of myostatin, TGF-β1 and pro-inflammatory cytokines plays a role in the lack of fibrotic tissue formation in injured hibernating squirrel muscle.

Additionally, we showed a clear activation of Wnt signaling at 3 and 6–8 weeks after CTX injury of muscles of hibernating squirrels, while no increase in canonical Wnt signaling was observed in injured muscles of summer animals. Previous analyses have shown that upregulation of Wnt signaling is important for later stages of muscle regeneration, whereas its downregulation is necessary in early stages of the muscle regeneration response. Furthermore, the lack of early inhibition of Wnt signaling can lead to fibrosis [38], [39], [40]. In contrast, other reports have shown that Wnt signaling enhances satellite cell proliferation [44], [69]. Interestingly, activation of the Wnt signaling pathway does not seem to promote fibrosis formation in regenerating hibernating animals. We speculate that Wnt signaling likely contributes to induction of proliferation of MPCs and differentiation of a sub-population of myocytes at later stages of differentiation.

In summary, our data demonstrate that active tissue turnover orchestrated by satellite cells does not likely contribute to the preservation of muscle mass during hibernation. Furthermore, despite evidence for a marked delay in muscle remodeling after infliction of injury, hibernating squirrels, unlike any other mammals, do not form fibrotic tissue and eventually repair muscle damage after emerging from hibernation. The molecular mechanisms underlying these observations represent a regulated interplay of inflammatory factors, p21, myostatin, MAPK and Wnt signaling. Therefore, we propose that hibernating squirrels are an interesting model system to study delayed muscle regeneration in the absence of fibrotic tissue formation that can be used to evaluate novel and existing therapeutic targets for preventing fibrosis and improving muscle regeneration during various stages of muscle injury.

## Materials and Methods

### 13-Lined ground squirrels (*Ictidomys tridecemlineatus*)

Most species of ground squirrels (Rodentia; Sciuridae) are capable of highly regulated hibernation in winter. Hibernation in ground squirrels formally includes 1-several week periods of torpor interspersed by 10–12 hour interbout arousals during which animals sleep (i.e. remain essentially immobile). Hibernation in mammals leads to profound reductions of blood flow, heart rate, metabolism, respiratory rate, and, in small hibernators, core body temperature [70]. These events do not represent a loss of homeostasis, but instead are precisely controlled and spontaneously reversible [71]. Hibernation enables some mammals to survive cold winter conditions when food and water are scarce and the demand for metabolic heat production is high. Although the energy savings of torpor are as large as 95%, there is some requirement for periodically exiting torpor (arousal) and resuming a high metabolic rate for a short period of time before re-entry into torpor.

In spring, squirrels emerge naturally from torpor and they return to high body temperatures (euthermia) and food and water intake is restored.

All experimental procedures were approved by the Animal Care and Use Committee of Johns Hopkins University School of Medicine and by the relevant IACUC, and followed federal welfare guidelines. Hibernation-naïve euthermic weanlings of both sexes were obtained from the captive breeding colony at the University of Wisconsin Oshkosh [72]. Animals were supplied with food and water *ad libitum* and monitored daily by animal cage inspection. During November/December, when first torpor was observed, food and water were removed and the squirrels were transferred to a dark hibernaculum maintained at ∼4°C for hibernation. “Hibernating” tissue samples were collected from torpid squirrels 3–4 months after immergence into torpor (n = 11). In April/May, squirrels emerged naturally from torpor and were returned to normal room temperature; food and water were reinstated. ”Summer” tissue samples were collected from aroused squirrels 1–2 months after emergence (n = 16). Euthanasia by decapitation was preceded by either injection with ketamine or inhalation of isoflurane.

### X-ray irradiation to remove resident stem cells

Squirrels were anesthetized with Ketamine and then confined in a plastic rat tube. The right hind limb was extended through a hole in the side of the tube and a single dose of 25.0 Gy ^137^Cs γ rays was administered using a J.L. Shepherd Mark I ^137^Cs irradiator (Glendale, CA) [19]. The dose rate was 5.5 Gy/min. The rest of the animal was shielded and protected by a secondary lead attenuator.

### Cardiotoxin treatment

To reproducibly create a skeletal muscle injury, 750 µl of 10 µM cardiotoxin (CTX) from *Naja nigricollis* (Calbiochem) were injected into squirrel gastrocnemius muscle. Both summer and hibernating squirrels were sacrificed at 4 days, 7 days, 2 weeks, 3 weeks, 6 weeks, 8 weeks and 10 weeks after CTX injection. Some CTX-injected hibernating squirrels were allowed to complete 6–10 weeks of hibernation and emerge naturally in spring; these were sacrificed 4 weeks after emergence.

### Histology and immunofluorescence

Muscle samples were rapidly processed by flash freezing and subsequently mounted in OCT Compound (Tissue-Tek, Sakura Finetek). For histology studies, embedded samples were sectioned into 10 µm sections using a cryostat (Microm HM 550). They were stained with hematoxylin and eosin or Masson's trichrome method following standard protocols [20]. For immunofluorescence studies, sections were first blocked with 10% goat serum, 5% bovine serum albumin, or 3% goat serum/5% bovine serum albumin for 1 hour at room temperature. They were next incubated in primary antibodies (anti-Dystrophin at 1∶300 from Abcam or anti-developmental myosin at 1∶150 from VECTOR) overnight at 4°C. After washing, they were incubated in secondary antibodies (Alexa Fluor 350, 488 and 594; 1∶400; Invitrogen) for 1 h at room temperature. After washing, sections were mounted on glass slides using Fluoromount-G Mounting Media (SouthernBiotech). All images were taken using a Nikon Eclipse 80i microscope.

### Morphometry

Skeletal muscle morphometry was performed following standard methods [20], [73] with some modifications. Fiber diameter and the minimal Feret's diameter were determined using Nikon's NIS elements BR3.0 software (Laboratory Imaging, Nikon) with a minimum of 2,000 fibers per animal.

### Immunoblot analysis

To determine protein expression, flash-frozen muscles were lysed with ice-cold lysis buffer (Nonidet P-40 1%, glycerol 10%, NaCl 137 mM, Tris-HCl pH 7.5 20 mM) containing protease (Complete Mini, EDTA-free, Roche) and phosphatase inhibitors (PhosSTOP, Roche). Then lysates were cleared by centrifugation at 14,000 rpm for 15 min at 4°C. Protein concentrations were determined with the Pierce BCA Protein Assay Kit (Thermo Scientific). Twenty µg of protein lysates were electrophoresed in Bis-Tris or Tris-glycine gels and transferred onto nitrocellulose membranes via standard procedures. Staining of proteins contained in membranes and polyacrylamide gels were carried out with Ponceau S Red (Sigma) and a Colloidal Blue Staining kit (Invitrogen), respectively. Membranes were incubated with primary antibody diluted in blocking solution (5% milk or 5% bovine serum albumin) overnight at 4°C. Primary antibodies used were: anti-Vinculin, anti-Desmin (Sigma); anti-Myogenin, anti-MyoD, anti-Myf5, anti-Myf6, anti-Myostatin (GDF-8/11 (F-13)), anti-p21 (Santa Cruz Biotechnology); anti-Phospho-Erk1/2, anti-Erk1/2, anti-Phospho-p38 MAPK, anti-p38 MAPK, anti-Phospho-ß-Catenin, anti-ß-Catenin, anti-p21, anti-TNF-α (Cell Signaling); anti-TGF beta 1, anti-IL6 (Abcam); anti Wnt-3a (R&D Systems); anti-Wnt16 (Epitomics). Immunoreactive bands were detected with HRP-conjugated secondary antibodies (Amersham) and visualized with Supersignal West Dura or Supersignal West Femto Substrates (Pierce).

### Statistical analysis

All values are expressed as mean ± s.e.m. Significance was determined by unpaired Student's t-tests. Significance was established at p≤0.05.

## Supporting Information

Figure S1 **Delayed skeletal muscle regeneration during hibernation.** Hibernating squirrels were injected with CTX and sacrificed at different time points after the injury. **A:** Representative pictures of H&E staining of gastrocnemius muscle from hibernating animals 6, 8 and 10 weeks after CTX injections. Hibernating animals exhibited delayed regeneration as compared to summer animals, but interestingly had an absence of fibrotic tissue even after 10 weeks without resolving inflammation. **B–E:** Developmental myosin immunofluorescence in summer (left) and torpid squirrels (right) revealed the time course of fiber regeneration. **C:** 8 weeks after CTX injury, regenerating cells are widespread in torpid animals. **D:** Summer squirrels displayed the first regenerating skeletal muscle fibers 2 weeks after cardiotoxin treatment, but not 4 d after injury (**B**). **E**: Regeneration is complete in muscle collected 4 weeks after CTX-injured hibernating animals aroused from torpor. CTX = cardiotoxin; d = days; w = weeks. N = 10. Bars represent 50 µm.
(EPS)

Figure S2 **Western blots from gastrocnemius muscles non-injected with cardiotoxin and integrity of the protein samples used for these analyses.** **A–D:** Western blot analysis from non-injected contralateral gastrocnemius muscles of summer and hibernating squirrels treated with cardiotoxin, and using antibodies against the proteins indicated. We did not see significant changes in the levels of the proteins analyzed between summer and hibernating animals. Samples are loaded in the same order for all blots. **E:** Staining with Colloidal Blue of proteins used for western blot analysis showing the integrity of the samples (see Methods S1). S: summer; H: hibernating; P = Phospho; d = days; w = weeks.
(EPS)

Methods S1 **Proteins used for Western blot analysis were stained both in gels and membranes in order to verify their integrity.**
(DOCX)
