# Supplementary material for: Impaired Skeletal Muscle Regeneration in the Absence of Fibrosis during Hibernation in 13-Lined Ground Squirrels
Source: PLoS One. 2012 Nov 14;7(11):e48884. doi: 10.1371/journal.pone.0048884 (PMC3498346; doi:10.1371/journal.pone.0048884)
Supplement: Methods S1 — Proteins used for Western blot analysis were stained both in gels and membranes in order to verify their integrity. (DOCX) [file pone.0048884.s003.docx]

**Supporting Information Methods S1**

**Protein staining**

We had issues with levels of expression of loading control proteins (see vinculin signal in Fig.6B) in 4 d and 3 w samples from hibernating animals treated with CTX, although we carefully loaded the same amount of protein (20 µg) in all wells. In order to verify the integrity and the amount of protein in the samples, we performed Ponceau S Red staining of proteins transferred to a nitrocellulose membrane, and Colloidal Blue staining of the same proteins contained in a polyacrylamide gel (Fig.S2E). Our analysis confirmed the integrity of our samples, and that we cannot use housekeeping genes for loading control in 4 d and 3 w CTX treated-samples because the toxin causes a massive damage of the tissue (due to long periods of inflammation, cell infiltration and edema). For this reason, to compare samples, we used the fixed amount of 20 µg protein loaded and staining of gels and membranes for verification.
